# No Difference in Weight Loss, Glucose, Lipids and Vitamin D of Eggs for Breakfast Compared with Cereal for Breakfast during Energy Restriction

**DOI:** 10.3390/ijerph17238827

**Published:** 2020-11-27

**Authors:** Jennifer B. Keogh, Peter M. Clifton

**Affiliations:** UniSA Clinical and Health Sciences, University of South Australia, Adelaide, SA 5000, Australia; peter.clifton@unisa.edu.au

**Keywords:** eggs, breakfast, energy intake, weight loss, overweight, obesity

## Abstract

The aim of the study was to investigate the impact of consuming 2 eggs for breakfast 5 days per week compared with eating breakfast cereal in a randomized parallel study. Two energy-restricted diets with a similar energy content were compared over a 6-month period. One hundred and ten participants—aged 56 ± 16 years, BMI 34 ± 6 kg·m^2^, 84 women and 26 men—commenced and 76 completed the study, 33 in the egg group and 43 in the cereal group. Weight loss in completers was 8.1 kg ± 7.0 kg (8.8 ± 6.4%) in the egg group and 7.3 kg ± 4.0 kg (7.6 ± 4.6%) in the cereal group (*p* < 0.001 for time) but there was no differential effect of diet (*p* = 0.56). Vitamin D was 55 ± 18 nmol/L at baseline rose at 3 months and fell at 6 months but remained higher than baseline (*p* < 0.001 for time) with no difference between the groups. Vitamin D levels were inversely correlated with BMI (r = −0.22 *p* = 0.025) and positively with age (r = 0.26 *p* = 0.009), and change in Vitamin D was positively correlated with weight change at 3 and 6 months (r = 0.46 and r = 0.41 both *p* < 0.001). In a post-hoc analysis of obese participants there was an effect of time (*p* < 0.01) and a time by diet interaction (*p* < 0.04), such that participants in the egg group maintained the increase in Vitamin D levels at 6-months. There was no effect on glucose and no adverse effects on total and LDL cholesterol, which did not change. In conclusion, both diets achieved clinically meaningful weight loss. There were no adverse effects on LDL-cholesterol, and there may be a beneficial effect on Vitamin D in people with obesity but this remains to be investigated in a prospective study.

## 1. Introduction

In 2017–2018, two thirds (67.0%) of Australian adults were overweight or obese (12.5 million people), an increase from 63.4% in 2014–2015 [1]. Many adults are trying to lose weight, as shown by a recent systematic review and meta-analysis of personal weight control attempts worldwide that estimated that 42% of adults reported trying to lose weight [2].

Eating breakfast can assist individuals to lose weight and maintain weight loss [3,4]. However, there are few recent data on the numbers of people who eat breakfast in Australia. In the Australian Health Survey (2011–2012), breakfast cereals were eaten by 36% of the population with a further 7% eating porridge [5]. Data from the 1995 National Nutrition Survey indicates that >77% of people ate breakfast but fewer than 10% had a cooked breakfast, with cereals, bread and milk being the most frequently eaten foods [6]. There are few studies examining the effects of an energy-restricted diet with eggs for breakfast on weight loss. In an 8-week study, greater weight loss of ~1 kg was reported in participants on an energy-restricted diet that included eggs but there was no effect on weight of including eggs without energy restriction [7]. However, this is a small difference over a short time period, and a longer duration needs to be explored as the finding may have occurred by chance alone. A potential benefit of eggs eaten at breakfast may be that they can help satisfy hunger and improve satiety. In an earlier study, we demonstrated that when eggs were eaten for breakfast there was a significantly reduced energy intake at lunch compared with a cereal breakfast with the same energy content (4518 ± 1593 vs. 5284 ± 1814, *p* = 0.001) [8].

Vitamin D deficiency has been well described in obese people, which may be due to a dilution effect of the greater volume of fat, liver and muscle [9]. Data from the 2011–2013 Australian Health Survey reported that 20% of adults (19% men; 21% women) were Vitamin D-deficient (<50 nmol/L) and 43% were classified as Vitamin D-insufficient (45% men; 42% women) ([10]). Adequate Vitamin D levels are important for bone health and increasing egg intake may improve Vitamin D status. Few foods contain Vitamin D and include fortified margarine, fatty fish such as salmon, herring and mackerel, and eggs [11]. Therefore, increasing egg intake may improve Vitamin D status. The Vitamin D content of eggs is 8.2 µg per serve (2 eggs—edible portion 104 g) or 7.9 µg per 100 g. As the adequate intake (AI) for adults (51–70 year) in Australia is 10 µg, one serving of eggs provides 82% of the AI [11].

The relationship between egg consumption and cardiovascular disease and type 2 diabetes remains a matter of debate [12]. There are few intervention studies examining the effects of eggs during energy restriction. Fuller (2018) found similar weight loss with no differences between groups in glucose control or lipids when 2 eggs for breakfast was compared with no eggs in a 12-month study in 128 participants with prediabetes or type 2 diabetes [13].

The aim of the study was to investigate the impact of an energy-restricted weight loss diet including 2 eggs for breakfast compared with eating cereal for breakfast on 5 days per week over a 6-month period on body weight, glucose and lipids, and Vitamin D status. The hypothesis was that the participants who regularly eat eggs for breakfast would have greater weight loss, increased lean mass preservation and better Vitamin D status relative to participants who regularly eat cereal for breakfast.

## 2. Materials and Methods

**Figure 1:**
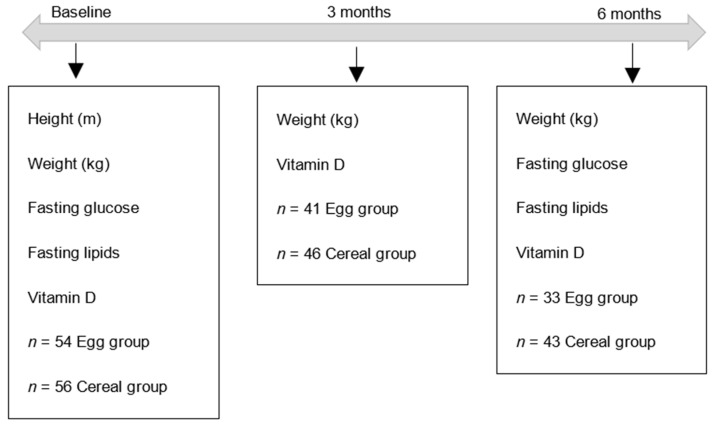
Flow chart of timeline, sample size at each timepoint and variables measured.

This was a randomised parallel study. The primary outcome of this study was weight loss. Secondary outcomes were fasting blood lipids, glucose and Vitamin D status (Figure 1).

Participants were >18 years, overweight (BMI > 25 kg/m^2^). Exclusion criteria were previous surgery for weight reduction; type 1 or type 2 diabetes; women who were or wished to become pregnant; women who were breast feeding; and participants reporting they were unwell or receiving medical treatment or were participating in any ongoing dietary studies. Participants self-reported their health status. Participants were given a $Au150 honorarium at the end of the study. The study was approved by the university’s Human Research Ethics Committee.

Based on the study by Noakes et al 2005, which had a weight loss of 7.3 kg (SD 3 kg), we expected we would be able to see a difference of 2 kg between groups (*p* < 0.5, 80% power) with 35 completers in each group [14].

A drop-out rate of 10–15% was anticipated, and 110 participants commenced so that 95–100 participants would complete the study. Participants were recruited using print and social media, the radio and advertising flyers on the university’s campuses.

Following initial contact with the Clinical Trial Facility, participants were asked to complete a Diet and Lifestyle Questionnaire to determine eligibility to participate in the study.

Participants attended the UniSA Clinical Trials Facility (City East campus) on 7 occasions over 6 months. Participants fasted from 8 pm the night before each visit, with only water permitted. At the first and last visits participants had weight, and height (once only), measured. A blood sample was taken for measurement of glucose, lipids and Vitamin D.

The energy restriction was 5500 kJ for women and 7000 kJ based on previous studies; this was an approximately 25–30% energy reduction [14,15]. The diets contained a variety of everyday foods, including eggs, bread, margarine, milk, fruit, meat, fish, chicken (or equivalent), vegetables and salad, yoghurt (or equivalent) and potato (or equivalent) for the egg group, or breakfast cereal, bread, margarine, milk, fruit, meat, fish, chicken (or equivalent), vegetables and salad, yoghurt (or equivalent) and potato (or equivalent) for the cereal group. The dietary intervention was explained by a qualified dietitian during the first visit. Participants were asked to keep daily checklists of their breakfasts during the study.

Compliance with both breakfasts was assessed from the compliance checklists completed by the participants. The cereal group were asked to avoid eating eggs for breakfast but could include eggs at other meals. Eggs and the breakfast cereal were provided for approximately half of the study until the advent of COVID-19. After this, vouchers were provided so individuals could purchase these foods during their usual grocery shopping for the remainder of the study.

Blood samples for fasting glucose, lipids and Vitamin D were collected and analyzed by a certified commercial laboratory (Clinpath Pathology, Mile End, SA 5031, Australia).

Results are expressed as mean ± SD unless otherwise stated. An Independent-Samples T-Test was carried out to compare the groups at baseline. Analysis of variance (ANOVA) with repeated measures was used to determine the effect of time and treatment at 3 and 6 months. Statistical analysis was performed using SPSS 24.0 (IBM, Armonk, NY, USA).

## 3. Results

One hundred and ten participants—56 ± 16 years, range 18–78 years, 84 women and 26 men—were randomised to commence the study *n* = 54 in the egg for breakfast group and *n* = 56 in the cereal for breakfast group. Baseline characteristics for all participants are presented in Table 1, and baseline characteristics for participants who completed the study are presented in Table 1**.** Baseline characteristics of non-obese and obese participants at baseline are shown in Table 2. Seventy-six participants completed the study. Withdrawals that were 21 for eggs and 13 for cereal were not statistically different (*p* = 0.1), giving a drop-out rate of 30%.

### 3.1. Weight and Vitamin D at 3 Months

Eighty-seven participants remained in the study at 3 months, 86 ± 18 kg, *n* = 41 egg group and 91 ± 16 kg cereal group, *n* = 46. Weight loss (5.2 ± 4.5 kg egg group versus 5.2 ± 2.8 kg cereal group) was not different between the groups (*p* = 0.948). Percent weight loss was 5.5 ± 4.0% in the egg group and 5.5 ± 2.8% in the cereal group. Vitamin D concentrations (64.5 ± 17.6 nmol/L, *n* = 38 egg group and 64.9 ± 18.2 nmol/L cereal group) were not different between the groups (*p* = 0.925).

### 3.2. Outcomes at 6 Months

Seventy-six participants completed the study (33 in the egg group and 43 in the cereal group). Weight loss in completers was 8.1 ± 7.0 kg (8.8 ± 6.4%) in the egg group and 7.3 kg ± 4.0 kg (7.6 ± 4.6%) in the cereal group (*p* < 0.001 for time). Repeated measures ANOVA showed a significant effect of time (*p* < 0.001) with a diet by time effect that was not significant (*p* = 0.56). Gender and baseline BMI had no significant effect when added as covariates. The egg group had two individuals with very large weight losses of >20 kg, which skewed the data. Removal of these two individuals normalised the data but had no effect on the results but the egg weight loss was lower at 6.7 ± 4.6 kg.

### 3.3. Fasting Glucose and Lipids

Glucose concentrations did not change (Table 3).

There was no change in total cholesterol, HDL-cholesterol 1, LDL cholesterol and triglyceride levels (Table 3).

### 3.4. Vitamin D

Mean Vitamin D levels were within the normal range at baseline (Table 4). Vitamin D rose at 3 months and fell at 6 months but remained higher than baseline (*p* < 0.001 for time). There was no difference between the two groups (*p* = 0.098 for diet by time). Vitamin D levels were inversely correlated with BMI (r = 0.22 *p* = 0.025) and positively with age (r = 0.26 *p* = 0.009), and change in Vitamin D was positively correlated with weight change at 3 and 6 months (r = 0.46 and r = 0.41 both *p* < 0.001). Vitamin D concentrations in participants who remained in the study at 3 months are shown in Table 3.

More than 70% of the participants were obese (79 of 110) with BMI 36.3 ± 5.8 kg/m^2^. In a post-hoc analysis when only obese participants were included in the analysis of Vitamin D, there was an effect of time (*p* < 0.01) and a time-by-diet interaction (*p* < 0.04), such that participants in the egg group maintained the increase in Vitamin D levels seen in both groups at 6-months (Table 5). Glucose, lipid and Vitamin D concentrations in non-obese and obese participants who completed the study at baseline, 3 months (Vitamin D only) and 6 months, Table 5.

### 3.5. Compliance

All 76 participants who completed the study completed the compliance checklists. Overall compliance to both breakfasts was approximately 95% assessed from compliance checklists completed by the participants.

## 4. Discussion

The main finding of this study was that eating two eggs for breakfast resulted in a similar weight loss compared with cereal for breakfast in an energy-restricted diet. Overall lipids and glucose did not change and there were no adverse effects on total or LDL cholesterol. Vitamin D levels rose at 3 months, declined at 6 months and remained higher than the baseline value.

Egg cholesterol is known to elevate LDL cholesterol to a small degree. A recent meta-analysis showed that increasing dietary cholesterol from a mean of 214 up to 821 mg/day (about three eggs) increased LDL cholesterol by about 0.2 mmol/L [16]. Thus, an increase of two eggs per day for 5 days per week might increase LDL cholesterol by 0.10 mmol/L, which would not be measurable, nor would it have any significant effect on cardiovascular disease risk. Most data on egg consumption and CVD events in normal subjects, except Li (2013) [17], has shown no relationship, although a recent paper [18] of a pooled analysis of six US studies showed each additional half an egg consumed per day was significantly associated with higher risk of incident CVD (adjusted HR, 1.06 [95% CI, 1.03–1.10]), and with all-cause mortality (adjusted HR, 1.08 [95% CI, 1.04–1.11]), which was very similar to that seen by Li [17]. A larger meta-analysis by Shin et al. found no relationship between egg consumption and CVD; nor was any seen in the Physicians Health Study, although a relationship with total mortality was noted [19,20]. On balance, the possible relationship between eggs and CVD risk remains unproven.

Participants enrolled in the study between September and December 2019. Therefore, participants commenced the study at the end of Winter, during Spring, and at the beginning of Summer. Vitamin D at 3 months was measured at the end of Spring at the beginning and end Summer or early Autumn. The changes in Vitamin D are likely to be related in part to the season in which it was measured and in part to weight loss. It is of interest that in a post-hoc analysis when only obese participants were included in the analysis of Vitamin D, participants on the egg diet maintained the increase in Vitamin D levels seen in both groups at 3-months. This remains to be clarified in a prospective study in obese participants only.

There are some limitations to the study that should be considered. There was a high drop rate of 30%, which may have been influenced by the advent of COVID-19. Longer studies often have a higher drop-out rate, and we have seen drop-out rates of 56% in a 12-month study and 67% by 24 months [21]. In contrast, in a 3-month weight loss study in people with type 2 diabetes we had a drop-out rate of 19% [22]. In the present study, 23 people (21%) had dropped out by three months.

The power analysis indicated that *n* = 35 were needed in both groups to see a difference in the primary outcome. In the end, *n* = 33 participants in the egg group and *n* = 43 cereal group completed the study, which may have limited our ability to detect a difference between the groups.

Dietary composition and energy intake were not assessed as food records were not kept. We were concerned that food records would increase attrition from the study as they impose an extra burden on participants and are often poorly completed.

The study was conducted in participants with normal glucose and lipids. Nevertheless, it is surprising the LDL cholesterol did not reduce given the 8% weight loss.

A strength of the study is that both eggs and the breakfast cereal were provided for approximately half of the study until the advent of COVID-19. Vouchers were provided so individuals could purchase these foods during their usual grocery shopping.

## 5. Conclusions

Both eggs and cereal eaten for breakfast during an energy-restricted diet resulted in similar weight loss. There were no adverse effects on total and LDL cholesterol. There may be a beneficial effect on Vitamin D in people with obesity but this remains to be investigated in a prospective study.

## Figures and Tables

**Table 1:** Baseline characteristics of participants who commenced the study and baseline characteristics of participants who completed the study ^1^.

	Commenced		Completed	
	Eggs (*n* = 54)	Cereal (*n* = 56)	Eggs (*n* = 33)	Cereal (*n* = 43)
Age years	54 ± 15	57 ± 15	57 ± 15	59 ± 15
Weight kg	94 ± 21	93 ± 18	90 ± 17	95 ± 16
BMI kg/m^2^	34 ± 7	34 ± 6	32 ± 5	34 ± 5
Glucose mmol/L	5.0 ± 0.7	4.9 ± 0.5	5.1 ± 0.7	5.0 ± 0.5
Total Cholesterol mmol/L	5.3 ± 0.9	5.5 ± 0.9	5.4 ± 0.8	5.4 ± 0.8
HDL-C mmol/L	1.5 ± 0.3	1.5 ± 0.3	1.5 ± 0.3	1.5 ± 0.3
LDL-C mmol/L	3.2 ± 0.8	3.5 ± 0.8	3.4 ± 0.7	3.4 ± 0.8
Triglycerides mmol/L	1.1 ± 0.4	1.3 ± 0.7	1.2 ± 0.4	1.2 ± 0.5
Vitamin D nmol/L	54 ± 19	56 ± 17	53 ± 15	57 ± 17

^1^ No significant differences observed; HDL = High density lipoprotein cholesterol; LDL = Low density lipoprotein cholesterol.

**Table 2:** Baseline characteristics of non-obese and obese participants at baseline who completed the study.

	Non-Obese		Obese	
	Eggs (*n* = 9)	Cereal (*n* = 11)	Eggs (*n* = 24)	Cereal (*n*= 32)
Age years	63 ± 14	48.6 ± 21.5	54.8 ± 15.4	62.2 ± 9.5 ^1^
Weight kg	75.4 ± 12.3	77.6 ± 12.1	95.8 ± 15.4	101.2 ± 12.9
BMI kg/m^2^	27.6 ± 1.7	27.7 ± 1.6	34.1 ± 4.3	36.0 ± 4.3
Glucose mmol/L	5.0 ± 0.4	4.8 ± 0.4	5.1 ± 0.8	5.0 ± 0.5
Total Cholesterol mmol/L	5.7 ± 1.0	5.4 ± 1.0	5.3 ± 0.7	5.4 ± 0.8
HDL-C mmol/L	1.7 ± 0.2	1.5 ± 0.3	1.5 ± 0.3	1.4 ± 0.3
LDL-C mmol/L	3.5 ± 0.8	3.4 ± 0.9	3.3 ± 0.7	3.4 ± 0.7
Triglycerides mmol/L	1.1 ± 0.5	1.2 ± 0.5	1.2 ± 0.4	1.2 ± 0.6
Vitamin D nmol/L	55.8 ± 13.0	57.9 ± 14.0	51.3 ± 16.1	56.9 ± 18.3

^1^*p* < 0.05 Participants in the cereal group who were obese were older that those in the egg group; BMI = Body mass index; HDL = High density lipoprotein cholesterol; LDL = Low density lipoprotein cholesterol.

**Table 3:** Glucose and lipid concentrations ^1^.

	Baseline		6 Months	
	Eggs	Cereal	Eggs	Cereal
Glucose mmol/L	5.1 ± 0.7	5.0 ± 0.8	5.1 ± 0.5	5.2 ± 0.7
Total Cholesterol mmol/L	5.4 ± 0.8	5.4 ± 0.8	5.4 ± 1.0	5.3 ± 0.9
LDL- C mmol/L	3.4 ± 0.7	3.4 ± 0.8	3.4 ± 0.7	3.3 ± 0.9
HDL- C mmol/L	1.5 ± 0.3	1.5 ± 0.3	1.5 ± 0.3	1.4 ± 0.3
Triglyceride mmol/L	1.2 ± 0.4	1.1 ± 0.4	1.2 ± 0.5	1.2± 0.5
Vitamin D (nmol/L) 1	52.9 ± 15.7	56.7 ± 17.0	58.3 ± 20.0	58.6 ± 1.4

^1^ No significant differences observed. Blood samples were available for *n* = 32 in the egg group and *n* = 41 in the cereal group; HDL = High density lipoprotein cholesterol; LDL = Low density lipoprotein cholesterol

**Table 4:** Vitamin D concentrations in participants in the study at 3 months (nmol/L) ^1^.

	Baseline	3 Months
Egg group (*n* = 38)	52.9 ± 15.7	64.5 ± 17.6
Cereal group (*n* = 44)	56.5 ± 17.2	64.9 ± 18.2

^1^*p* < 0.001 for time.

**Table 5:** Glucose, lipid and Vitamin D concentrations in non-obese (*n* = 9 egg group, *n* = 11 cereal group) and obese (*n* = 23 egg group, *n* = 30 cereal group) participants who completed the study at baseline, 3 months (Vitamin D only) and 6 months.

	Non-Obese						Obese					
	Baseline		3 Months		6 Months		Baseline		3 Months		6 Months	
	Eggs	Cereal	Eggs	Cereal	Eggs	Cereal	Eggs	Cereal	Eggs	Cereal	Eggs	Cereal
Glucose mmol/L	5.0 ± 0.4	4.8 ± 0.4			5.0 ± 0.4	5.1 ± 0.5	5.1 ± 0.8	5.0 ± 0.8			5.2 ± 0.6	5.2 ± 0.7
Total Cholesterol mmol/L	5.7 ± 1.0	5.4 ± 1.0			5.7 ± 1.2	5.1 ± 0.7	5.3 ± 0.7	5.4 ± 0.8			5.2 ± 0.8	5.4 ± 0.9
LDL- C mmol/L	3.5 ± 0.8	3.4 ± 0.9			3.5 ± 1.0	3.1 ± 0.8	3.3 ± 0.7	3.4 ± 0.7			3.3 ± 0.7	3.4 ± 0.8
HDL- C mmol/L	1.7 ± 0.2	1.5 ± 0.3			1.7 ± 0.2	1.4 ± 0.3	1.5 ± 0.3	1.5 ± 0.3			1.4 ± 0.3	1.5 ± 0.3
Triglyceride mmol/L	1.1 ± 0.5	1.2 ± 0.5			1.1 ± 0.5	1.1 ± 0.4	1.2 ± 0.4	1.2 ± 0.6			1.1 ± 0.4	1.2 ± 0.6
Vitamin D nmol/L	55.8 ± 13.0	57.9 ± 14.0	65.0 ± 12.1	64.6 ± 16.2	55.7 ± 13.3	63.7 ± 17.0	51.7 ± 16.8	56.2 ± 182	66.2 ±19.7	64.8 ± 17.6	59.3 ± 22.2	56.7 ± 17.4

HDL = High density lipoprotein cholesterol; LDL = Low density lipoprotein cholesterol.
